# Emotional intelligence and perceived stress in healthcare students: a multi-institutional, multi-professional survey

**DOI:** 10.1186/1472-6920-9-61

**Published:** 2009-09-17

**Authors:** Yvonne Birks, Jean McKendree, Ian Watt

**Affiliations:** 1 Department of Health Sciences, University of York, York, UK; 2 Hull York Medical School, York, UK

## Abstract

**Background:**

Emotional intelligence (EI) is increasingly discussed as having a potential role in medicine, nursing, and other healthcare disciplines, both for personal mental health and professional practice. Stress has been identified as being high for students in healthcare courses. This study investigated whether EI and stress differed among students in four health professions (dental, nursing, graduate mental health workers, medical) and whether there was evidence that EI might serve as a buffer for stress.

**Method:**

The Schutte Emotional Intelligence and the Perceived Stress scale instruments were administered to four groups of healthcare students in their first year of study in both the autumn and summer terms of the 2005-6 academic year. The groups were undergraduate dental, nursing and medical students, and postgraduate mental health workers.

**Results:**

No significant differences were found between males and females nor among professional groups for the EI measure. Dental students reported significantly higher stress than medical students. EI was found to be only moderately stable in test-retest scores. Some evidence was found for EI as a possible factor in mediating stress. Students in different health profession courses did not show significant differences in Emotional Intelligence.

**Conclusion:**

While stress and EI showed a moderate relationship, results of this study do not allow the direction of relationship to be determined. The limitations and further research questions raised in this study are discussed along with the need for refinement of the EI construct and measures, particularly if Emotional Intelligence were to be considered as a possible selection criterion, as has been suggested by some authors.

## Background

Emotional intelligence (EI) is increasingly discussed as having a potential role in medicine, nursing, and other healthcare disciplines, both for personal mental health and professional practice. Stress has been identified as being high for students in healthcare courses. This study investigated whether measures of EI and stress differed among students in four health professions (dental, nursing, graduate mental health workers, medical) and whether there was evidence that EI might serve as a buffer for stress.

The concept of stress has been widely discussed in relation to healthcare students and reports of high levels of perceived stress amongst these groups are common [1-3]. All students experience the demands of course work, a new environment and new people, and for those living away from home for the first time learning to manage financially, emotionally and socially by themselves. In addition, healthcare students, encounter other potential sources of stress such as the emotions involved in dealing with patients and the learning of applied clinical skills [1,4]. Stress in healthcare students has been associated with increased levels of depression [5,6] use of drugs and alcohol and increased anxiety [3] and attrition [7,8].

The expense involved in training healthcare professionals represents a considerable investment and attrition has a significant financial impact as well as being unfortunate for the student involved. It would therefore seem important to identify those students who may experience their course as more stressful than their peers in order to target them early for help and support.

Several predictors of stress in healthcare students have been identified in previous literature. While some are concrete problems such as childcare arrangements [9], financial security [10] and volume of work [11], there is a body of literature which points to a range of individual psychological characteristics as predictive of stress in students regardless of other mitigating circumstances [9,12,13].

One such factor, emotional intelligence (EI), is increasingly made reference to in medicine, nursing and other healthcare disciplines where it is suggested it is important for professional mental health as well as effective practice [2,14-16]. The concept of EI was introduced over a decade ago by Salovey and Mayer [17] and is described as 'a type of social intelligence that involves the ability to monitor one's own and other's emotions, to discriminate among them, and to use this information to guide one's thinking and actions'. It emerged from an array of research looking at how people perceive, communicate, and use emotions.

Popular or public interest in EI arose from a book by Goleman [18] which suggested that life success depended more on emotional intelligence than cognitive intelligence. As is often the case in an emerging area, the use of a variety of terms makes it difficult to agree on an over-arching definition of EI and it has been referred to as emotional literacy, the emotional quotient, personal intelligence, social intelligence, and interpersonal intelligence [19]. One of the most rigorous examinations of EI to date (a meta-analysis of the relationship between EI and performance outcomes) suggests that EI is "the set of abilities (verbal and nonverbal) that enable a person to generate, recognize, express, understand, and evaluate their own, and others, emotions in order to guide thinking and action that successfully cope with environmental demands and pressures" [20].

Recent calls have been made to include training in emotional intelligence in healthcare workers as a means of improving leadership qualities, preventing burnout and stress, and improving curricula and communication skills. It is cited in various literature as ' essential' for nurse managers' [21], nursing and medical recruitment [22,15] and curricula [23] however, little empirical work has examined EI in health professionals or its impact on professional and academic outcomes. The few studies examining this so far have demonstrated that EI was positively associated with lower perceived stress in dental undergraduates [2] and a short intervention to raise awareness of emotional intelligence has been reported but not evaluated prospectively [16]. Current evidence for variation in professionals and ways in which it might be effectively included in curricula or continuing professional development is lacking [24].

This paper reports a study looking at emotional intelligence across four healthcare student groups in their first year of study and examines the relationship with perceived stress. While it is important to understand stress and coping mechanisms across the curriculum for this study first year students were chosen because, if the results warrant, the students can be followed through the curriculum for a longitudinal study and if the results are promising, these measures could be used in the future to identify students early in the course who may be particularly highly stressed or low in EI and might benefit from additional support.

## Methods

The study was conducted with four groups of healthcare students in their first year of study in both the autumn and summer terms of the 2005-6 academic year. Ethical approval was granted by the Department of Health Sciences Ethics Committee at the University of York and by the Medical Education Ethics Committee at Hull York Medical School. Students who commenced the first year of their programme in the academic year 2005-6 were approached in the Autumn term of 2005 to take part in the study. Dental students were attending Barts and the London School of Medicine and Dentistry, Queen Mary's College, University of London. Medical students were from the Hull York Medical School. Diploma nursing students were attending the Department of Health Sciences at the University of York as were the postgraduate students who were studying the area of mental health with a view to becoming post-graduate mental health workers in primary care. Repeat questionnaires were given again toward the end of the Summer term in 2006. No exclusion criteria were applied but students were self-selecting as participation was entirely voluntary.

All students received an information sheet explaining the study and three questionnaires for completion. One questionnaire collected demographic information, one measure of Emotional Intelligence and one measure of Perceived Stress. Students either completed the questionnaire immediately after the lecture or returned the questionnaires in pre-paid postal envelopes.

Emotional intelligence was measured using a scale developed by Schutte et al. [25]. The scale comprises 33 items, three of which are reversed scored. Participants are required to rate the extent they agree or disagree with each statement on a five-point scale (1 = strongly disagree; 5 = strongly agree). Recent factor analytic studies by the scale authors have established that all the items load significantly on a single factor. The score is calculated by summing the item responses.

Stress was measured using the Perceived Stress Scale [26]. This 10 item scale was developed to measure the degree to which individuals appraise their life as stressful and has been widely used in health studies. Four of the items are reversed score and the scale has a 5-point Likert response format. The total score is calculated by summing responses. The PSS was designed for use with community samples with at least a junior high school education. The questions are general in nature and relatively free of content specific to any sub population group.

Demographic information was also collected using a questionnaire developed by the authors for this purpose. Analysis was performed using SPSS version 16.

## Results and discussion

Baseline data collection provided data from 68 of 109 (62%) dental students, 100 of 134 (75%) medical students, 104 of 114 (91%) nursing students, and 17 of 21 (81%) graduate mental health students. Numbers at follow up for those who completed all the questionnaires at both times were substantially reduced with only 25 dental students, 43 medical students, 64 nursing students and 15 graduate mental health students completing both the first and the second set of questionnaires. Any missing items not completed by the participants were replaced by the median for that item. There were no missing items for the PS scales and a total of 0.35% of items overall on the EI scales. No participant in the group completing all questionnaires had more than 1 missing item at Time 1 or Time 2. The demographic characteristics of the groups at baseline are summarised in Table 1.

**Table 1:** Demographic Characteristics of Student Groups at Time 1

	Medical	Nursing	Mental Health	Dental
**Gender (%)**				

Male	45 (45%)	12 (11.5%)	4 (23.5%)	24 (35.3%)

Female	55 (55%)	92 (88.5%)	13 (76.5%)	44 (64.7%

				

**Age**				

Mean (SD)	22.07 (5.20)	27.08 (8.76)	25.65 (7.23)	20.44 (3.42)

Range	18-42	18-50	21-46	18-40

				

**Ethnicity (%)**				

White	71 (71%)	97 (93.3%)	14 (82.4%)	10 (14.7)

Black or British black	6 (6%)	4 (3.8%)	1 (5.9%)	9 (13.2)

Asian or British asian	17 (17%)	1 (1%)	1 (5.9%)	35 (51.5%)

Mixed	1 (1%)	0	0	1 (1.5%)

Chinese or other ethnic group	3 (3%)	0	0	12 (17.6%)

Missing	2 (2%)	2 (1.9%)	0	1 (1.5%)

The mean scores and 95% confidence intervals for the Schutte Emotional Intelligence Scale (EI) and Perceived Stress Scale (PS) for students completing any of the instruments at each individual time period (called "full sample" in the table) and the subset of 147 students that filled in all measures at both times ("subset" in the table) are presented in Table 2.

**Table 2:** Mean Emotional Intelligence (EI) and Perceived Stress (PS) scores at Time 1 and Time 2

	Dental	Medical	Mental Health	Nursing
**EI Time 1 Full Sample** **(95% CI)**	122.6 N = 68 (119.5-125.5)	124.7 N = 100 (122.2-127.1)	126.5 N = 17 (120.5-132.4)	124.6 N = 104 (122.1-127.0)

**EI Time 1 Subset** **(95% CI)**	122.3 N = 25 (117.7-126.9)	128.0 N = 43 (124.5-131.5)	126.7 N = 15 (120.8-132.6)	124.1 N = 64 (121.2-126.9)

**EI Time 2 Full Sample** **(95% CI)**	124.8 N = 25 (120.1-129.5)	125.6 N = 44 (122.3-128.6)	126.6 N = 16 (122.0-133.8)	123.4 N = 66 (121.0-125.7)

**EI Time 2 Subset** **(95% CI)**	124.8 N = 25 (120.3-129.3)	125.9 N = 43 (122.5-129.4)	126.7 N = 15 (122.4-134.1)	124.6 N = 64 (121.8-127.4)

**PS Time 1 Full Sample** **(95% CI)**	16.5 N = 68 (12.0-18.0)	15.5 N = 100 (14.3-16.7)	16.7 N = 17 (13.7-19.6)	15.9 N = 104 (14.7-17.1)

**PS Time 1 Subset** **(95% CI)**	16.3 N = 25 (14.0-18.6)	14.5 N = 43 (12.7-16.3)	16.5 N = 15 (13.5-19.5)	15.3 N = 64 (13.8-16.7)

**PS Time 2 Full Sample** **(95% CI)**	19.3 N = 35 (17.3-21.3)	15.1 N = 44 (13.4-16.5)	18.1 N = 16 (15.1-21.1)	17.8 N = 67 (16.3-18.7)

**PS Time 2 Subset** **(95% CI)**	19.4 N = 25 (17.1-21.8)	15.1 N = 43 (13.3-16.9)	18.5 N = 15 (15.5-21.6)	17.9 N = 64 (16.4-19.4)

Mean Emotional Intelligence (EI) and Perceived Stress (PS) scores for full sample at each time and subset of participants completing all EI and PS scales at both Time 1 and Time 2

Scores for total EI can range from 33 to 165 and for PS between 0 and 40. Reliability analyses were conducted by calculating Cronbach's alpha for the 33-item Schutte emotional intelligence scale (N = 289; alpha = 0.87) and 10-item Perceived Stress Scale (N = 289; alpha = 0.85) using the data from all participants who completed each scale at Time 1. This indicated that the internal reliability of each scale was adequate for further analysis.

The scores for only those 147 participants who completed both scales at baseline and follow up provided the complete data on which all subsequent analyses were carried out. Reliabilities for both scales were identical to the full group for this subset who completed all scales.

The correlation for Emotional Intelligence at baseline and follow up was r = .65 which would indicate a relatively stable trait over time, as measured by the Schutte EI scale. A paired t-test for EI at Time 1 and Time 2 indeed showed no significant difference in the EI scores over time, (t = -.08, p = .94). This would support the scale authors' assertion that it is a stable trait, although the correlation indicates that it may be somewhat variable over time.

The correlation for Perceived Stress from Time 1 to Time 2 was r = .46, and a paired t-test for the group overall did show a significant change in stress over time with the group as a whole showing higher stress at Time 2 (t = -3.97, p < .0001). This second administration of the measures was close to end of year exams which may account for the increase in stress at this date. This choice allowed evaluation of whether there is a mitigating effect of Emotional Intelligence on Perceived Stress at times that are traditionally stressful for students.

### Differences between student groups

**Figure 1:**
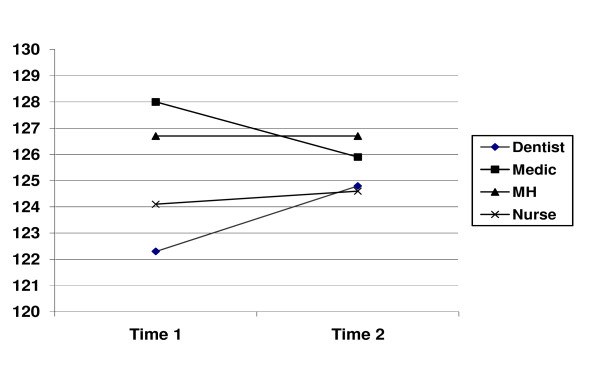
**Emotional Intelligence scores at Time 1 and Time 2 for participants completing all scales**.

Analysis of differences by groups using analysis of variance showed no differences among professional categories for Emotional Intelligence (EI Time 1: F = 1.67, p = .179; EI Time 2: F = .24, p = .87). The highest scoring group at Time 1 (medical students) showed a reduction in EI and the lowest scoring group (dental students) an increase. 95% confidence intervals, given in Table 2, suggest that the groups overlap significantly due to high variance which may indicate regression to the mean in terms of changes in scores. This pattern can be seen in Figure 1.

A similar analysis for Perceived Stress showed no difference between groups (F = .70, p = .55) at baseline. However, at Time 2, there was a significant difference between groups (F = 3.41, p = .02). A post hoc comparison using the Scheffe test which adjusts for multiple comparisons indicated that the only statistically significant difference was between the medical students and the dental students at Time 2, as shown in Table 3.

**Table 3:** Post hoc comparison (Sheffé test) of professional groups on Perceived Stress at Time 2

Professional Group		Mean Difference	Significance
medic	Nurse	-2.78	.14
	MH	-3.44	.30
	dentist	-4.35*	.04*

nurse	MH	-.66	.99
	dentist	-1.57	.75

MH	dentist	-.91	.98

*Indicates a significant difference

**Figure 2:**
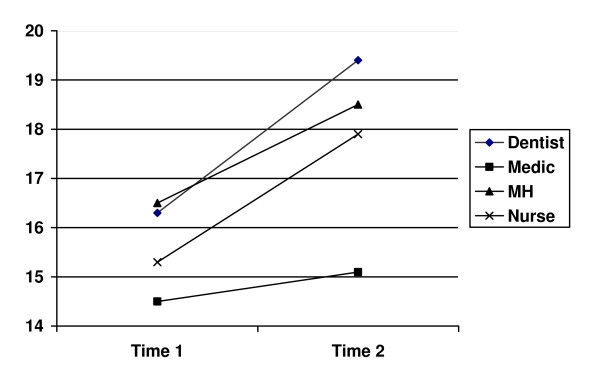
**Perceived Stress scores at Time 1 and Time 2 for participants completing all scales**.

The paired t-test looking at differences between Time 1 and Time 2 showed a significant increase in stress over time, though a one-way ANOVA did not indicate differences among the professional groups. Figure 2 demonstrates that all groups increased their mean score on PS from baseline to follow-up.

### Differences in Age and Gender

A one-way ANOVA indicated a significant difference between the groups for average age (F = 6.47, p < .0001). Comparing the groups using the Scheffe test for post hoc planned comparisons showed that nursing students were marginally older on average than the medical students (mean difference = 3.9 years, p = .06) and significantly older than the dental students (mean difference = 7.0 years, p < .001). However, the correlation between Emotional Intelligence and age for the whole group was non-significant (r = .07) as was the correlation between Perceived Stress and age (r = -.03 at Time 1; r = .07 at Time 2). Therefore, no further analysis was carried out using age as a variable.

The means with the sample split by gender for those who completed all four scales were also examined. Samples size for males in some categories (5 nurses of 64, 3 mental health students of 15) were too small for reliable analyses between student groups. Comparing males and females on EI and on PS at Time 1 and Time 2 demonstrated no difference in scores by gender for either measure.

Given that levels of emotional intelligence did not differ significantly between ages, gender or student group, subsequent analyses were carried out on the group as a whole.

### Emotional Intelligence and Stress

The correlation between Mean EI and PS at Time 1 was r = -.27 (p < .001) and at PS Time 2, r = -.22, p = .007. The significant negative correlation indicates that those with higher EI have lower PS at baseline and follow-up. The slightly reduced correlation at Time 2 may indicate that while EI might help moderate stress at lower levels, when there is an acute stressor such as end of year exams, the effect of EI may be lessened.

The mean change in EI and PS over time varied for participants. The average change in EI score (.06 points) was low, considering that the scores can range from 33 to 165, indicating a reasonably stable trait in the group overall. However, looking at the maximum drop in EI (-31 points) and the maximum gain in EI (28 points), it is clear that some individuals showed considerable variation in their scores, up to approximately a 20% change in EI. The changes in PS varied from -22 points to 14 points with a mean change of -2.

The correlation of the change in the measures (EI1-EI2 and PS1-PS2) was r = -.39, p < .0001. Therefore those students whose change in EI increased had a significant decrease in their perceived stress and vice versa. The constraints of correlational tests mean we are unable to specify any causal direction to this relationship.

## Conclusion

This study examined the hypothesised link between perceived stress and emotional intelligence in a variety of healthcare students. Previous work had suggested a link between EI and perceived stress in student populations.

Emotional Intelligence in this context appears to be at some level a moderator of stress. However its effect seems to be slightly less pronounced at time two where generally higher levels of stress were reported. The reason for the higher levels of stress was not formally identified but many of the groups had upcoming exams which may have contributed to increased stress. Given that the question of interest was whether high EI may help students cope with stress, the measures at a time of average versus high stress is a useful feature of this dataset. Nevertheless, because of the correlational nature of the study, we cannot conclude the direction of any causal connection. It may be that as people get more stressed, their EI scores decrease, or that as EI scores decrease for whatever reason, stress increases. However, given that EI is more stable than PS, it might make sense to hypothesise that it is EI that is affecting stress rather than the other way around. There may be important individual differences in the behaviour and stability of EI that would certainly warrant further investigation.

Dental students were more stressed toward the end of the first year of study than the medical students. This dental cohort differed in that it had the youngest mean age and was more ethnically diverse than any of the other groups and this may have contributed to higher levels of perceived stress. However, low numbers particularly for dental students in the follow up stage of the research made this impossible to follow up meaningfully in this study.

The study suggests that Emotional Intelligence seems to be a relatively stable construct as measured using the Schutte scale and as claimed by the originators of the scale [25], while perceived stress, rather unsurprisingly, varies significantly at different times. While there is variability in the EI scores, this would be expected even if EI is a relatively stable trait because of error of measurement in the test. Nevertheless, other measures of EI may yield more accurate, stable or informative information and should be compared to the Schutte scale used here.

This study is preliminary and the sample size is small, primarily because of the low return rate at Time 2. Nevertheless, even looking at only those students who completed all scales at both times, some interesting results were found. There is some indication that Emotional Intelligence is relatively stable over time, though it would useful to compare different measures of EI, including both trait and performance measures, to see if this holds true with different conceptualisations and measures of EI.

It is interesting that there were no gender, age or disciplinary group differences in EI scores for this group of health care students as this is not the case for other studies using different populations. Petrides and Furnham [27] demonstrated higher self estimated EI in males than females which in turn correlated with measured scores. Conversely, some studies have found females score higher in EI [28]. A significant developmental increase in social and emotional competencies from early adulthood to middle age has also been suggested by others [29,30]. Bar-On, R., 2000. Emotional and social intelligence: Insights from the emotional quotient inventory (EQ-i). In: Bar-On, R. and Parker, J.D.A., Editors, 2000. Handbook of emotional intelligence, Jossey-Bass, San Francisco, CA, pp. 363-388. However the continued use of different instruments from different theoretical conceptualisations of EI makes such results difficult to compare. It would be useful to repeat this study with larger numbers and a variety of student groups to determine whether professions in healthcare attract students with similar EI scores and how those may or may not differ from other groups. There is little literature with which to reference the student scores found in this study however the scores reported in the original scale validation paper for the EI measure cited mean scores for therapists and prisoners. The students' scores in this study were higher than those of prisoners and less than those of practicing therapists.

While this study suggests the link between EI and stress may be worth pursuing, much work remains to be done to fully explore the relationships between emotional intelligence and stress in students in various health professions and this study raises some interesting questions for further research.

One limitation of the present study is that it is based on correlational rather than experimental evidence, a limitation inherent in many studies of personal attributes. Further work will be required to determine how EI impacts on stress, and also on adaptation or coping and whether interventions may facilitate development of effective strategies.

Another potential limitation is that the EI variables in this study might significantly overlap with other variables not included in the study, which would suggest that EI may not be a distinctive measure. There is work which suggests EI is distinct from a wide variety of other measures, including the big five personality factors, self-esteem, trait anxiety, verbal and performance intelligence, and other well established measures [31]. However, it may be that other individual differences can account for variance in performance and stress attributed to EI. Since studies often use different conceptualisations of EI it makes definitive conclusions difficult. Other traits, for example, neuroticism or anxiety, may be confounded with reports of perceived stress. Thus, future work should control for other personality traits potentially associated with the Perceived Stress Scale.

EI research is still in its infancy, and further research is needed before we can fully understand the role that EI might play in moderating stress or other outcomes. Future work may develop the suggestion that higher EI may be associated with lower perceived stress by investigating whether teaching EI might increase feelings of control and competence. If EI skills can be developed then this should lead, in turn, to more effective coping, and better psychological adaptation.

## Competing interests

The authors declare that they have no competing interests.

## Authors' contributions

YB initiated the study, wrote the literature review and collected the data for the nursing, mental health and medical students. JM organised the medical student data collection and conducted the statistical analysis. Both contributed to writing the paper. IW supervised the project, commented on drafts and advised on design. All authors read and approved the final manuscript.

## Pre-publication history

The pre-publication history for this paper can be accessed here:

http://www.biomedcentral.com/1472-6920/9/61/prepub
